# Analysis of scientific collaboration in Chinese psychiatry research

**DOI:** 10.1186/s12888-016-0870-1

**Published:** 2016-05-26

**Authors:** Ying Wu, Xing Jin

**Affiliations:** School of Humanities and Social Sciences, Shanxi Medical University, No. 56, South Xinjian Road, Taiyuan, China; Affiliated Tumor Hospital, Shanxi Medical University, Taiyuan, 030013 China

**Keywords:** Psychiatry, Scientific collaboration, China

## Abstract

**Background:**

In recent decades, China has changed profoundly, becoming the country with the world’s second-largest economy. The proportion of the Chinese population suffering from mental disorder has grown in parallel with the rapid economic development, as social stresses have increased. The aim of this study is to shed light on the status of collaborations in the Chinese psychiatry field, of which there is currently limited research.

**Methods:**

We sampled 16,224 publications (2003-2012) from 10 core psychiatry journals from Chinese National Knowledge Infrastructure (CNKI) and WanFang Database. We used various social network analysis (SNA) methods such as centrality analysis, and Core-Periphery analysis to study collaboration. We also used hierarchical clustering analysis in this study.

**Results:**

From 2003-2012, there were increasing collaborations at the level of authors, institutions and regions in the Chinese psychiatry field. Geographically, these collaborations were distributed unevenly. The 100 most prolific authors and institutions and 32 regions were used to construct the collaboration map, from which we detected the core author, institution and region. Collaborative behavior was affected by economic development.

**Conclusion:**

We should encourage collaborative behavior in the Chinese psychiatry field, as this facilitates knowledge distribution, resource sharing and information acquisition. Collaboration has also helped the field narrow its current research focus, providing further evidence to inform policymakers to fund research in order to tackle the increase in mental disorder facing modern China.

## Background

China is now the world’s second largest economy country, having seen profound changes in recent decades. The proportion of the Chinese population suffering from mental disorder has grown in parallel with the rapid economic development, as social stresses have increased. According to recent data from the World Health Organization (WHO), the burden of mental disorder is highest, surpassing that of cardiovascular disease, respiratory system disease and malignancy [1]. As it drew the attention of Chinese researchers studying psychiatry, the prevention and control of mental disorder became a huge challenge for them to overcome. Since this field permeates several arenas of biomedicine, no single individual is trained to perform all specialty tasks. Thus, scientific collaboration, which improves communication, facilitates sharing of expertise, and provides opportunities for the emergence of new scientific ideas, is indispensable for the growth of the field of psychiatry in modern China.

There has been increased collaboration within and between different scientific fields over the last decade. Co-authorship is a frequently used and reliable measure of research collaboration [2]. In 2001, American researcher Newman used Social network analysis (SNA) to study on the structure of scientific collaboration networks in fields such as biomedicine, physics and computer science [3–5]. German scholar Kretschmer applied the method of science, information metrology and psychology in scientific collaboration skillfully and acquired a series of achievements by using SNA [6, 7]. SNA has been used to study collaboration in bibliographic co-authorship networks [8, 9]. A social network is defined as a set of social entities, such as people, organizations, and countries, with some pattern of relationship between them [10]. These networks are usually modeled by graphs, where nodes represent the social entities and lines represent the ties established between them. The underlying structure of such networks is the object of study of SNA.

There has, however, been a lack of publications on scientific collaboration within the Chinese psychiatry field. Therefore, this study was designed to measure the activities of this field at the micro level (authors), meso level (institutions) and macro level (regions).

## Methods

We collected our data from 10 major journals in China National Knowledge Infrastructure (CNKI) and WanFang Database (2003 to 2012). Together, CNKI and WanFang Database cover the majority of journals from China. Each bibliographic record includes information such as the title, author names, abstract and key words. The 10 major journals include: (1) Journal of Clinical Mental Psychiatry, (2) Chinese Journal of Nervous and Mental Disease, (3) Journal of Neuroscience, (4) Journal of Clinical Psychosomatic Diseases, (5) Journal of Psychiatry, (6) Shang Hai Archives of Psychiatry, (7) Si Chuan Mental Health, (8) Chinese Journal of Psychiatry, (9) Journal of Neurology and Neurorehabilitation, and (10) Journal of International Psychiatry (Table 1). We believe that these 10 publications are sufficient to determine the structure of collaboration in the field of Chinese psychiatry research.

**Table 1:** Ten representative Journals in Chinese psychiatry field

Journal title	Number of papers
Journal of Clinical Mental Psychiatry	2805
Chinese Journal of Nervous and Mental Disease	2598
Journal of Neuroscience	2147
Journal of Clinical Psychosomatic Diseases	1979
Journal of Psychiatry	1756
Shang Hai Archives of Psychiatry	1449
Si Chuan Mental Health	1442
Chinese Journal of Psychiatry	1098
Journal of Neurology and Neurorehabilitation	732
Journal of International Psychiatry	618

**Table 2:** The full names and acronyms of institutions

Name of Institution	Acronyms	Name of Institution	Acronyms
Anhui Medical University	AHMU	Shandong Jining Municipal Hospital for Mental Disease Prevention and Control	SD JN Hosp.
Anhui Mental Health Center	AH MHC	Shandong Liaocheng No. 4 People's Hospital	SD LC No. 4 Hosp.
Beijing Ankang Hospital	BJ Ankang Hosp.	Shandong Linyi Mental Health Center	SD LY MHC
Beijing Huilongguan Hospital	BJ HLG Hosp.	Shandong Mental Health Center	SD MHC
Beijing Neurology Consultation Center	BJ NCC	Shandong Qingdao Mental Health Center	QD MHC
Capital Medical University	CMU	Shandong University	SDMU
Central South University	CSU	Shandong Yantai Psychological Convalescent Hospital	SD YT Psych.Hosp.
Chongqing Medical University	CQMU	Shanghai Baoshan District Mental Health Center	SH BS MHC
Chongqing Mental Health Center	CQ MHC	Shanghai Changning District Mental Health Center	SH CN MHC
Foshan No.3 People's Hospital	FS No.3 Hosp.	Shanghai Hongkou District Mental Health Center	SH HK MHC
Fudan University	FU	Shanghai Jiao Tong University	SJTU
Guangdong Foshan No.3 People's Hospital	GD FS No.3 Hosp.	Shanghai Mental Health Center	SHH MHC
Guangdong Shantou No.4 People's Hospital	ST No.4 Hosp.	Shanghai Pudong New District Mental Health Center	SH PD MHC
Guangxi Longquanshan Hospital	GX LQS Hosp.	Shanghai University of Traditional Chinese Medicine	SHUTCM
Guangxi Medical University	GXMU	Shanghai YangPu District Mental Health Center	SH YP MHC
Guangxi Nanning No.8 People's Hospital	NN No.8 Hosp.	Shanghai Zhabei District Mental Health Center	SH ZB MHC
Guangzhou Brain Hospital	GZ Brain Hosp.	Shantou University	STU
Guangzhou Psychiatric Hospital	GZ Psych. Hosp.	Shenzhen Kangning Hospital	SZ Kangning Hosp.
Hangzhou No.7 People's Hospital	HZ No.7 Hosp.	Shenzhen Mental Health Institute	SZ MHI
Hangzhou Police Station Ankang University	HZ Ankang Univ.	Sichuan Mianyang Mental Health Center	SC MY MHC
Hebei Medical University	HBMU	Sichuan Panzhihua No.3 People's Hospital	SC PZH No.3Hosp.
Hebei Mental Health Center	HB MHC	Sichuan University	SCU
Hebei No.6 People's Hospital	HB No.6 Hosp.	Southern Medical University	STMU
Hebei Rongjun Hospital	HB Rongjun Hosp.	Sun Yat-sen University	SYSU
Henan Coal Health School	HN Coal Heal. Sch.	Suzhou Guangji Hospital	SZ GN Hosp.
Henan Psychiatric Hospital	HN Psych. Hosp.	Suzhou University	SZU
Huizhou No.2 People's Hospital	HZ No.2 Hosp.	Tianjin Anding Hospital	TJ Anding Hosp.
Institute of Forensic Science.Ministry of Justice P.R. China	IOFC.MOJ	Tianjin Ankang Hospital	TJ Ankang Hosp.
Jiangsu Wuxi Mental Health Center	JS WX MHC	Tianjin Medical University	TJMU
Jiangsu Yangzhou Wutaishan Hospital	YZ WTS Hosp.	Tongji University	TJU
Jiangsu Zhenjiang No.4 People's Hospital	ZJ No.4 Hosp.	Wuhan University	WHU
Kunming Medical University	KMMU	Xian Mental Health Center	XA MHC
Liaoning Shenyang Mental Health Center	SY MHC	Xinxiang Central Hospital	XX Central Hosp.
Luoyang No.5 People's Hospital	LY No.5 Hosp.	Xinxiang Medical University	XXMU
Nanjing Medical University	NJMU	Yunnan Psychiatric Hospital	YN Psych. Hosp.
Peking University	PKU	Zhejiang Huzhou No.3 People's Hospital	HZ No.3 Hosp.
Shandong Ankang Hospital	SD Ankang Hosp.	Zhengzhou No.8 People's Hospital	ZZ No.8 Hosp.
Shandong Binzhou People's Hospital	SD BZ Hosp.		

Centrality, which reflects status and rights of activities in their social network, is one of the most important metrics in network analysis. There are three common measures of centrality: degree centrality, betweenness centrality and closeness centrality [11]. In collaborative networks, if there is a direct connection between an actor and others, the actor is in the central position with greater rights. Degree centrality is equal to the number of nodes that connect with a central node. If an author/institution/region has the highest degree centrality, it is considered a central author/institution/region in the collaboration network [12]. In collaborative networks, if an actor is between two points, that actor is in the important posiition. Betweenness centrality is the number of shortest paths that pass through a given node [13]. In our study, having the highest betweenness centrality would indicate that the author/institution/region possesses and controls a great deal of resources for research. Finally, in collaborative networks, if all paths between an actor and others respectively are the shortest, this actor is in the core position. Closeness centrality of a given node is equal to the reciprocal of the total distance from this node to all other nodes. Thus, the closer a node is to all other nodes, the higher its closeness centrality. Having the lowest closeness centrality indicates that the author/institution/region is at the core of the entire network.

In Core-Periphery structure analysis, the network is divided into two arears--core area and periphery area. The nodes which are in the core area are in the important position.

Hierarchical clustering, which creates a hierarchy of clusters that can be represented by a dendrogram, has been used to extract subgroups from the co-authorship network in many studies. In this tree structure, the root of the tree consists of a single cluster containing all authors, while the leaves correspond to specific individuals.

SNA was used to analyze the collaboration structure of Chinese psychiatry research. We then used the UCINET program and Netdraw to produce a visual representation of the network. In addition, the software for frequency analysis was SATI (Statistical Analysis toolkit For Informetrics) published by Zhe Jiang University in China (Http://sati.liuqiyuan.com/#sati).

Firstly, we imported all the data into SATI and produced the co-occurrence matrix. Then, the information of the matrix was imported into UCINET to analysis of centrality, hierarchical clustering and core-periphery structure. Finally, we imported them to Netdraw to the analysis of visualization and formed the mapping knowledge domain.

In order to elucidate the main co-authorship structure of the network, we selected the 100 most prolific authors, the top 100 institutions and 32 regions from 2003 to 2012. This threshold resulted in the top 100 prolific authors who must have published 32 co-authorship papers to be included (two authors who have not collaborated with others were deleted, so the co-authorship map, a visualization of authors’ collaboration network, includes 98 authors). The top 100 institutions appeared more than 24 times (25 institutions which have not collaborated with others were deleted, so the collaborative map, a visualization of institutions’ collaboration network, includes 75 institutions) and there were 32 regions (there were no collaborations from Tibet, so it was excluded, leaving us with 31 regions).

## Results

We retrieved 16,224 papers spanning 2003-2012 from the ten psychiatry journals. Among those, 13,669 were co-authored. The percentage of co-authored papers rose from 78 to 80 % over the last 10 years, suggesting an increase in scientific collaboration in the Chinese psychiatry field.

### Analysis on collaboration at the micro (authors) level

Of all the publications from the Chinese psychiatry field in 2003 to 2012, more than 80 % were published by two or more collaborators and the output of achievements in scientific research by way of collaboration was consistent with the total output. This suggested that most published research was collaborative. In this study, the map was composed of 5 independent sub-networks. The line value and the distance between two vertices represent collaborative strength, while thickness of the line represents the number of co-authorship papers. In this authors’ collaboration network, the researcher working in Shanghai Mental center has the highest degree centrality of 33, indicating that with 33 collaborators, he was the most key author. In a collaboration network, betweeness centrality reflects the role that the author plays within the network. Without the author with the highest betweeness centrality, the collaborative network would be disrupted. Since the researcher working in Nanjing Medical University was the author with the highest betweeness centrality, he played an instrumental role in maintaining the cohesion of the network and thus had the power to influence collaborative relationships. In a collaborative network, the closer two authors are to each other, the more easily information is communicated and the more likely the two are to collaborate. The researcher working in Shanghai Mental health center had the lowest closeness centrality, indicating that he contributed a great deal of research and was in a central position within the entire network.

Using hierarchical clustering analysis, we divided the 98 authors into 5 sub-networks. The largest sub-network included 90 nodes and 1132 lines. In this sub-network, the average path length was 2.495 and the average clustering coefficient was 3.288, indicating a clustering effect. A researcher, who is in this sub-network, worked in the Shanghai Mental Health Center and had the highest centrality degree mainly due to his research focusing on neuroimaging of patients with mental disorders. The researcher, who also worked in the Shanghai Mental Health Center, had the second highest centrality degree mainly due to his research focusing on the neuropathology of mental disorders. The researcher, with the third highest centrality degree, mainly researched treatments for schizophrenia. The second sub-network included two researchers, who both worked in the Tian shui Mental Hospital of Gansu and researched pharmacological treatments of depression. The third sub-network included the researcher from the Fourth Hospital of Linyi in Shandong and the researcher from the Linyi Mental Health Center of Shandong, and their research focused on the classification of depression. The fourth sub-network included the researcher from Anhui Medical University and the researcher from The Fourth People’s Hospital of Hefei in Anhui, and they researched the neurophysiology of mental disorders. Finally, the fifth sub-network included two researchers from Xuanwu Hospital in Beijing, where they researched neurology.

### Analysis on collaboration at the meso (institutions) level

**Fig. 1:**
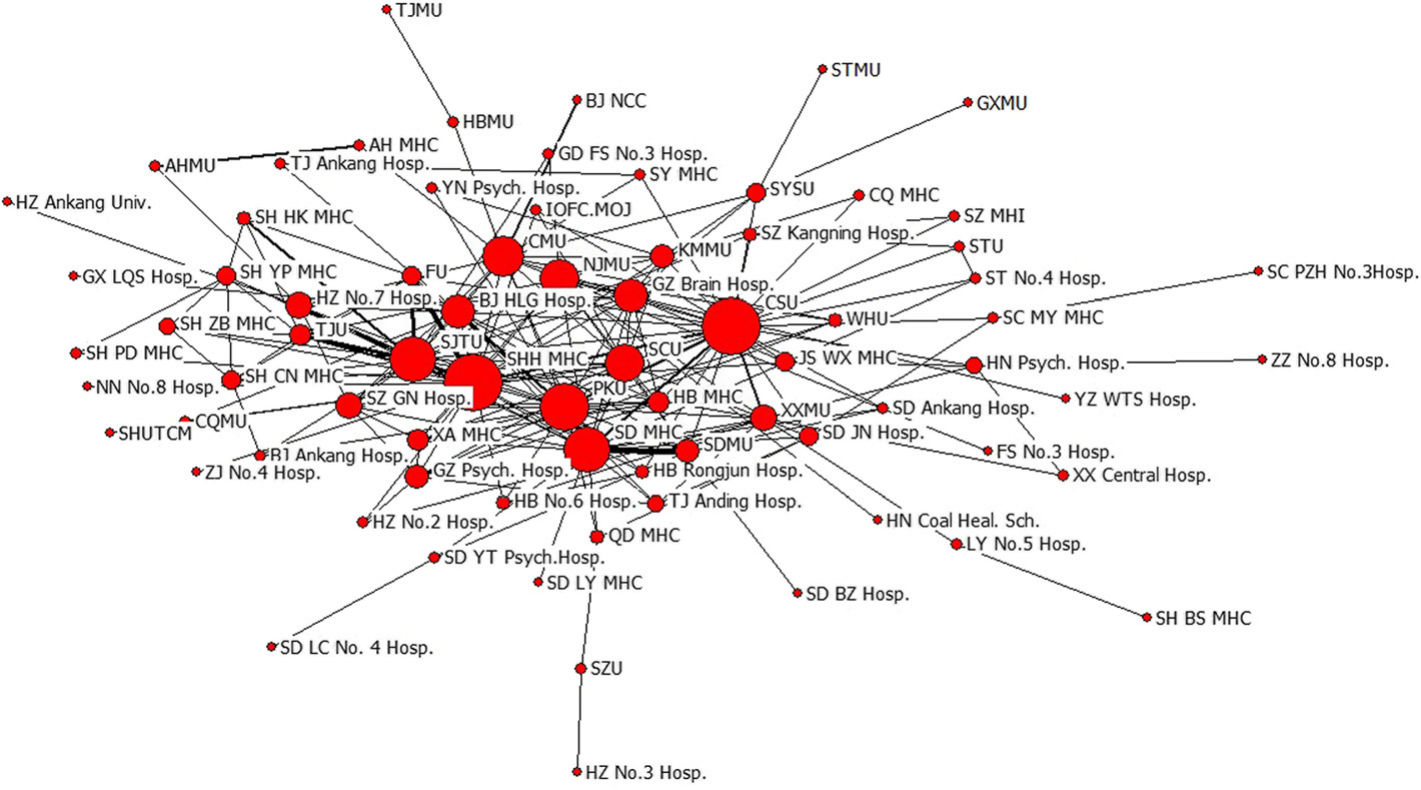
The structure map of the institutional collaboration network on Chinese psychiatry research (The geographic name and the name of university adopted the official abbreviation, see Table 2)

**Fig. 2:**
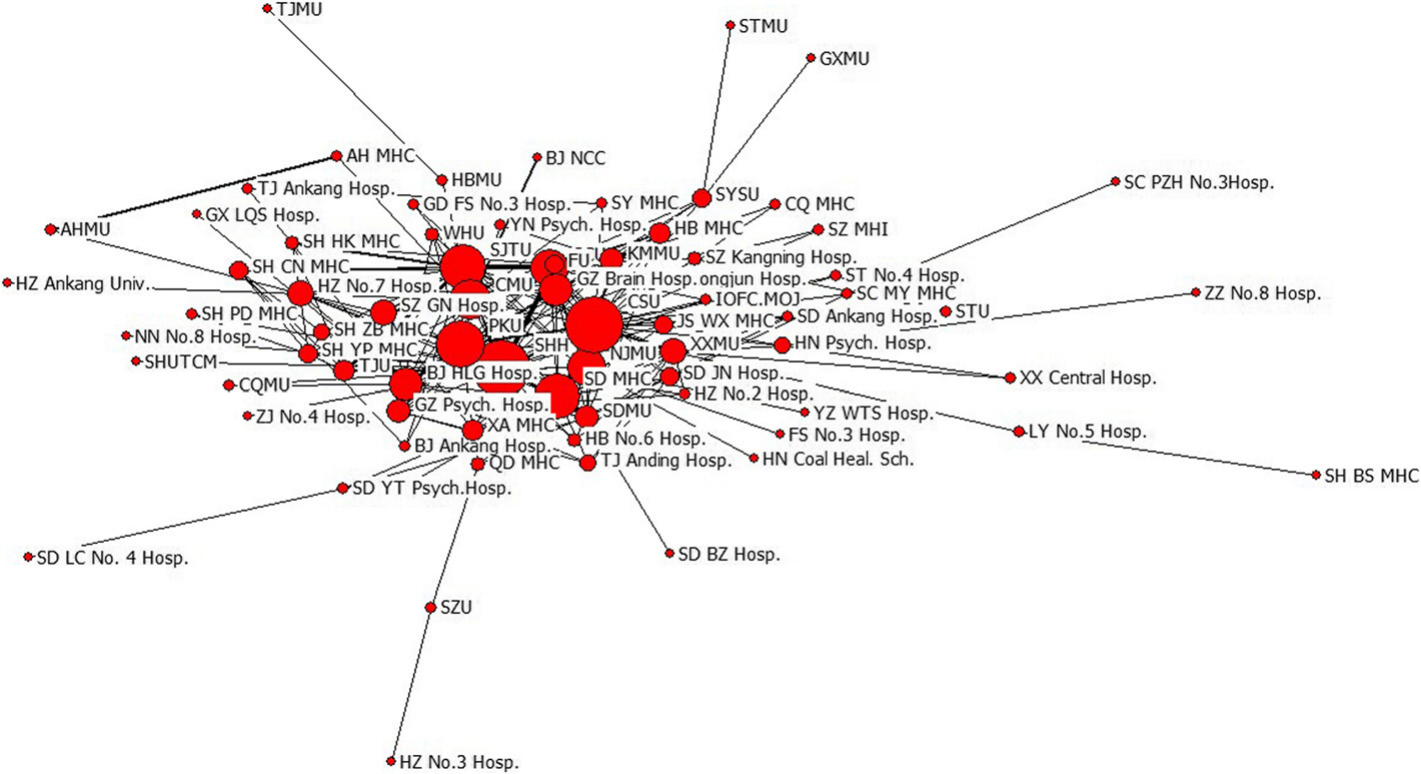
The core-periphery structure map of the institutional collaboration network on Chinese psychiatry research (The geographic name and the name of university adopted the official abbreviation, see Table 2)

Papers with multi-institutional collaborators accounted for more than half of all papers. Universities, research institutions and hospitals were the main institutions conducting research in this field. As collaborations among institutions increased, the total output in terms of scientific publications also increased from 2003 to 2012. In this study, there were 7,672 papers from SCI which demonstrated inter-institution collaboration from 2003 to 2012. This number increased from 418 in 2003 to 587 in 2012. These papers included 8,382 institutions that appeared a total of 32,410 times. The largest collaboration in our sample involved 20 institutions. We formed a map visualizing the structure of institutions’ collaboration network in the field of psychiatry during 2003 to 2012 (Fig. 1), the abbreviations of the geographic name and the name of university were listed in Table 2. The size of the node represents centrality in collaborative network. Shandong Mental Health Center and Mental Health Center of the Second Xiangya Hospital in Central South University had the highest degree centrality and betweenness centrality, and the lowest closeness centrality (Table 3). This indicated that these institutions were highly collaborative. The distance and thickness of the line between two nodes represents collaborative strength and the number of collaborative papers, respectively. We found that Shandong Mental Health Center, Mental Health Center of the second Xiangya Hospital in Central South University and Mental Health Center of Peking University were in the center of the collaborative network and thus played an influential role in the development of psychiatry. In contrast, The eighth People’s Hospital of Zhenzhou and The Fourth People’s Hospital of Liaocheng in Shandong were on the edge of the collaborative network. Compared to that of those institutions in the center, the scientific research strength of institutions on the edge were slightly weaker. Further, collaborations among the institutions in the center reflect ‘the center of the obvious effect’ in the process of co-authorship while the institutions on the edge collaborated more loosely. Analyzing Core-Periphery was a quantitative way to study networks. We applied this method to the collaboration network and found that the ‘correlative value of collaborative network’ was 0.698 (UCINET 6.0). A well-delineated Core-Periphery structure appeared (Fig. 2), indicating that the collaborative network was regional, whereby research institutions in close geographical proximity were more likely to collaborate, the abbreviations of the geographic name and the name of university were listed in Table 2. Shandong Mental Health Center and Shandong University, with the greatest number of collaborations, had a close collaborative research relationship.

**Table 3:** Top 10 institutions on centrality measures in collaborative network

Degree	Score	Betweeness	Score	Closeness	Score
Shanghai Mental Health Center	106	Central South University	1450	Central South University	130
Shanghai Jiaotong University	100	Shanghai Mental Health Center	1094	Shanghai Mental Health Center	136
Central South University	98	Shandong Mental Health Center	776	Shandong Mental Health Center	136
Shandong Mental Health Center	92	Capital Medical University	764	Shanghai Jiaotong University	140
Fudan University	65	Nanjing Medical University	747	Peking University	140
Peking University	58	Peking University	568	Capital Medical University	143
Shandong University	58	Shanghai Jiaotong University	555	Nanjing Medical University	146
Tongji University	42	Xinxiang Medical University	524	Guangzhou Brain Hospital	148
Capital Medical University	40	Beijing Huilongguan Hispital	376	Sichuan University	150
Xinxiang Medical University	33	Sun Yat-sen University	294	Beijing Huilongguan Hispital	152

### Analysis on collaboration at the macro (regions) level

**Fig. 3:**
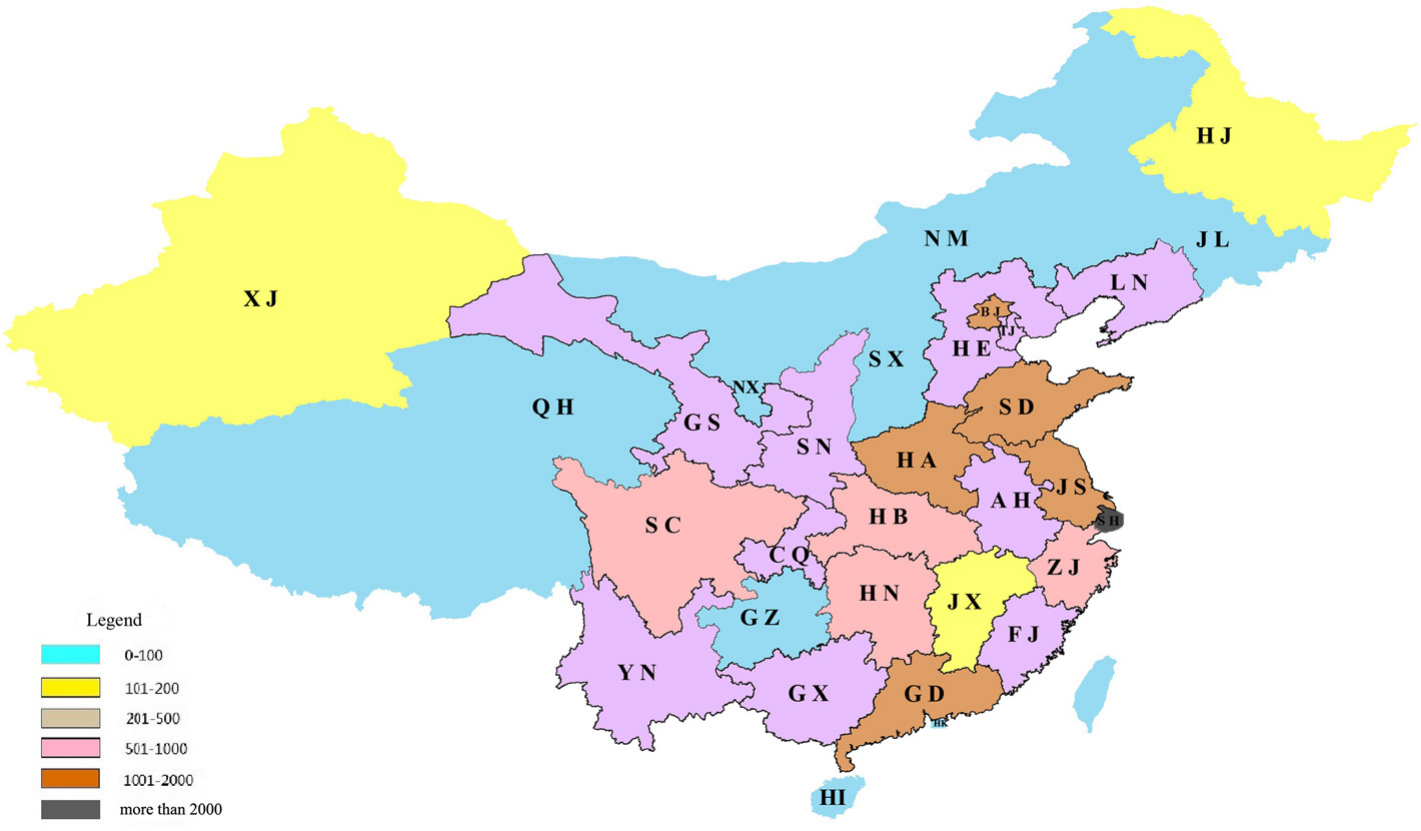
Regions distribution of Chinese psychiatric papers (The geographic name adopted the official abbreviation, see Table 4)

**Fig. 4:**
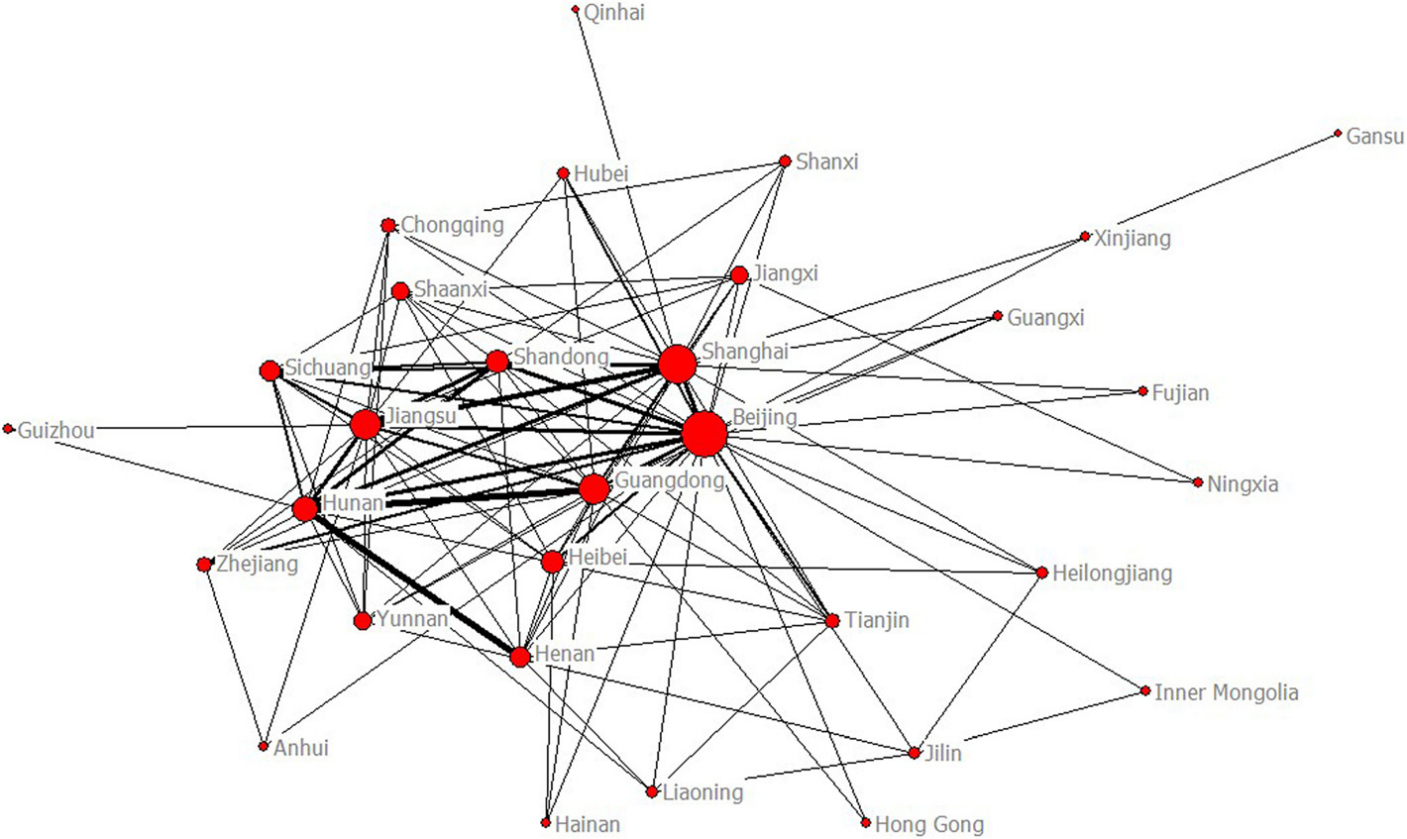
The structure map of collaboration network among regions on Chinese psychiatry research

**Fig. 5:**
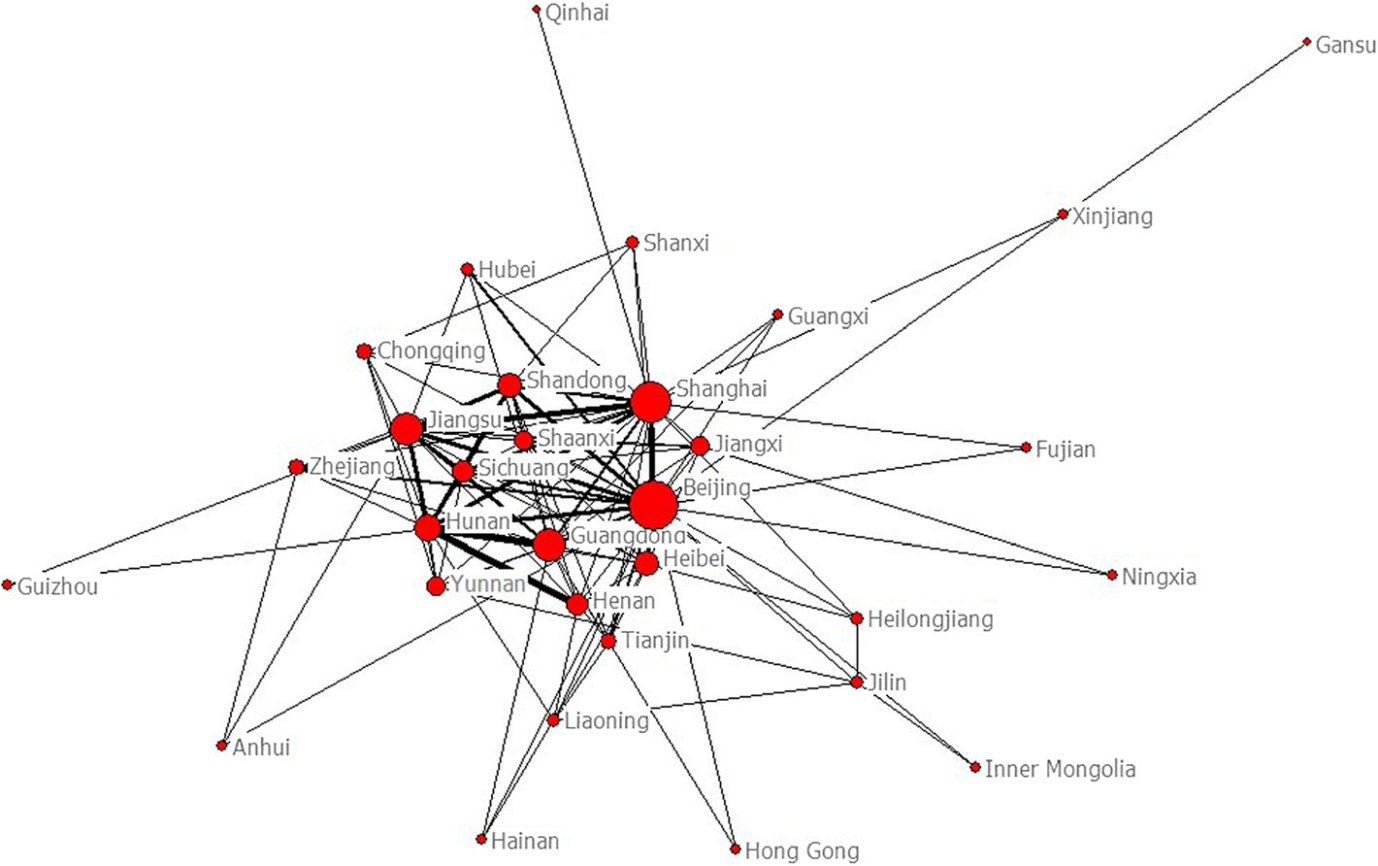
The core-periphery structure map of collaboration network among regions on Chinese psychiatry research

From 2003 to 2012, the most productive regions were Shanghai, Beijing and Shandong (Fig. 3), with 2,154 psychiatric research papers originated from Shanghai, making up 13.07 % of all such papers. Using the top 31 most productive regions in China to construct the map of scientific collaboration (Fig. 4), the network included 31 nodes and 226 lines. The average path length was 1.858 and the average clustering coefficient was 5.363, indicating a pronounced clustering effect. We applied Core-Periphery analysis and calculated the correlative value of the collaborative network to be 0.904 (UCINET 6.0). Once again, a well-delineated Core-Periphery structure appeared (Fig. 5).

We analyzed the effect of scientific collaboration on regions’ scientific output by correlating nodes to their corresponding scientific research achievements. For each of the 31 regions, we examined the number of ties and number of papers produced (Table 4). We found that the quantity of international collaborations correlates with research output. This suggested that international scientific collaboration greatly influenced scientific output in this field, whereby countries that collaborated frequently had a greater research output.

**Table 4:** The relation between scientific collaboration and papers

Collaboration		Regions	Production	
Ranks	Ties		Papers	Ranks
1	27	Beijing(BJ)	1214	6
2	21	Shanghai(SH)	2154	1
3	17	Guangdong(GD)	1495	4
4	16	Jiangsu(JS)	1688	3
5	13	Hunan(HN)	584	8
6	12	Hebei(HE)	303	15
7	11	Shandong(SD)	1754	2
8	10	Sichuan(SC)	555	9
9	9	Henan(HA)	1248	5
10	8	Yunnan(YN)	368	12
11	8	Shaanxi(SN)	284	16
12	8	Jiangxi(JX)	140	22
13	7	Zhejiang(ZJ)	620	7
14	7	Tianjing(TJ)	360	13
15	6	Chongqing(CQ)	270	17
16	5	Liaoning(LN)	305	14
17	5	Jilin(JL)	77	24
18	4	Hubei(HB)	529	10
19	4	Heilongjiang(HJ)	177	21
20	4	Shanxi(SX)	76	25
21	3	Anhui(AH)	369	11
22	3	Guangxi(GX)	267	18
23	3	Xinjiang(XJ)	107	23
24	3	Hainan(HI)	21	29
25	2	Fujian(FJ)	245	19
26	2	Ningxia(NX)	54	26
27	2	Guizhou(GZ)	53	27
28	2	Neimenggu(NM)	42	28
29	2	HongKong(HK)	10	31
30	1	Gansu(GS)	237	20
31	1	Qinghai(QH)	21	30

A centrality analysis (Table 5) revealed that Beijing, with the highest degree centrality of 120, highest betweenness centrality of 177, and lowest closeness degree of 90, was the center of the scientific collaboration network of China’s psychiatry field. Beijing’s extensive research output made it a major producer of international publications.

**Table 5:** Top 10 regions of China on centrality measures in collaborative network

Degree	Score	Betweeness	Score	Closeness	Score
Beijing	120	Beijing	177	Beijing	33
Hunan	112	Shanghai	83	Shanghai	39
Jiangsu	86	Jiangsu	29	Guangdong	44
Shanghai	81	Xinjiang	29	Jiangsu	45
Guangdong	60	Guangdong	26	Hunan	48
Shandong	52	Hunan	18	Hebei	49
Henan	38	Hebei	8	Shandong	50
Sichuan	30	Shandong	4	Sichuan	51
Hebei	25	Jiangxi	3	Henan	52
Tianjing	17	Yunnan	3	Yunnan	53

## Discussion

As China’s economy has grown and social pressures have increased, the prevalence of mental disorder has also grown dramatically. Because of the diversity and complexity of diseases this encompasses, scientific collaboration is indispensable if progress is to be made in the treatment of mental disorder. Although several studies have shown that collaboration has increased at the level of authors, institutions and regions [14–16], few have reported this phenomenon within the psychiatry field. This study retrieved bibliographic data of Chinese psychiatry research from 2003 to 2012 from CNKI and WanFang Database. We constructed and analyzed the structure of scientific collaboration at the micro (authors), meso (institutions) and macro (regions) levels based on SNA and found that scientific collaboration was correlated with this field’s development.

The authors who had the highest centrality were the central authors of the whole network, suggesting that they heavily influenced research in the Chinese psychiatry field and were thus leader of the field. In the era of a knowledge-based economy, as the most important economic factors, intellectual resources become more obviously valuable. Thus, scientific collaboration played a large role in the emergence of a subject leader.

The institutions which had the highest centrality were the center of multi-institutional collaboration in the Chinese psychiatry field possessing and controlling a great deal of resources for research. From the analysis on Core-Periphery structure, collaborating academic institutions obviously demonstrated ‘center effect’, while those collaborating with famous institutions demonstrated ‘elite institutions assembling’. In other words, the phenomenon that institutional collaborations was within the same country showed geographical characteristics. Thus, the other research institutions need to collaborate with institutions which collaborated closely to strive for the more scientific research resource.

At a regional level, Beijing had the highest centrality and was thus in the most central position. Our analysis suggested that regional scientific collaboration was positively correlated with total output in terms of scientific research. From the analysis on Core-Periphery structure, developed cities such as Beijing and Shanghai collaborated closely. China’s rapid economic development, which has encouraged collaborative behavior, has contributed to increases in research output. Higher-income regions prefer to collaborate with each other while lower-income regions prefer to collaborate with higher-income regions in order to increase the quality of their research.

## Conclusion

This study described the collaborative behaviors of research in the Chinese psychiatry field at the micro (authors), meso (institutions) and macro (regions) levels. Based on the centralities of the author ranking, academic leaders will be selected more easily. Furthermore, studying these collaborations not only can help researchers to master the forefront of this field but also provide scientific evidences and suggestions for policymakers to guide and manage the Chinese psychiatry field in the future.

## Abbreviations

AH, Anhui; AH MHC, Anhui Mental Health Center; AHMU, Anhui Medical University; BJ, Beijing; BJ Ankang Hosp., Beijing Ankang Hospital; BJ, HLG Hosp., Beijing Huilongguan Hospital; BJ NCC, Beijing Neurology Consultation Center; CMU, Capital Medical University; CNKI, China National Knowledge Infrastructure; CQ, Chongqing; CQ MHC, Chongqing Mental Health Center; CQMU, Chongqing Medical University; CSU, Central South University; FJ, Fujian; FS No.3 Hosp., Foshan No.3 People's Hospital; FU, Fudan University; GD, Guangdong; GD FS No.3 Hosp., Guangdong Foshan No.3 People's Hospital; GS, Gansu; GX, Guangxi; GX LQS Hosp., Guangxi Longquanshan Hospital; GXMU, Guangxi Medical University; GZ, Guizhou; GZ Brain Hosp., Guangzhou Brain Hospital; GZ Psych. Hosp., Guangzhou Psychiatric Hospital; HA, Henan; HB, Hubei; HB MHC, Hebei Mental Health Center; HB No.6 Hosp., Hebei No.6 People's Hospital; HB Rongjun Hosp., Hebei Rongjun Hospital; HBMU, Hebei Medical University; HE, Hebei; HI, Hainan; HJ, Heilongjiang; HK, HongKong; HN, Hunan; HN Coal Heal. Sch., Henan Coal Health School; HN Psych. Hosp., Henan Psychiatric Hospital; HZ Ankang Univ., Hangzhou Police Station Ankang University; HZ No.2 Hosp., Huizhou No.2 People’s Hospital; HZ No.7 Hosp., Hangzhou No.7 People's Hospital; IOFC.MOJ, Institute of Forensic Science.Ministry of Justice P.R.China; JL, Jilin; JS, Jiangsu; JS WX MHC, Jiangsu Wuxi Mental Health Center; JX, Jiangxi; KMMU, Kunming Medical University; LN, Liaoning; LY No.5 Hosp., Luoyang No.5 People’s Hospital; NJMU, Nanjing Medical University; NM, Neimenggu; NN No.8 Hosp., Guangxi Nanning No.8 People’s Hospital; NX, Ningxia; PKU, Peking University; QH, Qinghai; SATI, Statistical Analysis Toolkit For Informetrics; SC, Sichuan; SD, Shandong; SD Ankang Hosp., Shandong Ankang Hospital; SD BZ Hosp., Shandong Binzhou People's Hospital; SH, Shanghai; SN, Shaanxi; SNA, Social Network Analysis; ST No.4 Hosp., Guangdong Shantou No.4 People’s Hospital; SX, Shanxi; SY MHC, Liaoning Shenyang Mental Health Center; TJ, Tianjing; WHO, World Health Organization; XJ, Xinjiang; YN, Yunnan; YZ WTS Hosp., Jiangsu Yangzhou Wutaishan Hospital; ZJ, Zhejiang; ZJ No.4 Hosp., Jiangsu Zhenjiang No.4 People’s Hospital.
